# First experiences with PET-MRI/CT in radiotherapy planning for cervical cancer

**DOI:** 10.1007/s00404-022-06395-6

**Published:** 2022-03-09

**Authors:** Sophia Scharl, Clara-Bianca Weidenbaecher, Christine Hugo, Christine E. Brambs, Karina Knorr, Stephanie E. Combs, Lars Schüttrumpf

**Affiliations:** 1 Department of Radiation Oncology, Technische Universität München (TUM), Ismaninger Straße 22, Munich, Germany; 2 Department of Radiation Oncology, University Hospital Ulm, Albert-Einstein-Allee 23, 89081 Ulm, Germany; 3 Deutsches Konsortium Für Translationale Krebsforschung (DKTK), Partner Site Munich, Munich, Germany; 4 Institute of Radiation Medicine (IRM), Helmholtz Zentrum München, Ingolstädter Landstraße 1, Neuherberg, Germany; 5 Department of Obstetrics and Gynecology, Technische Universität München (TUM), Ismaninger Straße 22, Munich, Germany; 6 Department of Nuclear Medicine, Technische Universität München (TUM), Ismaninger Straße 22, Munich, Germany

**Keywords:** PET-MRI, Cervical cancer, Radiotherapy, Target volume

## Abstract

**Purpose:**

PET-CT has recently been included in the NCCN staging recommendations for cervical cancer stages II–IV and is already routinely applied to radiotherapy planning for other malignancies, as it is expected to provide higher accuracy for the detection of areas with tumor cell spread. In this study, we report on our first experiences of PET-based radiotherapy planning for cervical cancer.

**Methods:**

19 patients with cervical cancer that underwent pre-therapeutic PET imaging treated at our institution between January 2016 and April 2019 were included in the study. Information on the primary tumor, lymph node involvement, metastatic spread and changes in the radiotherapy procedure based on the PET findings are described.

**Results:**

A previously unknown primary tumor extension that was detected by PET imaging in one patient. In patients who underwent a PET before the systematic pelvic and paraaortic lymphonodectomy (*n* = 2), PET was false negative for pelvic lymph node metastases in 50%. In patients who underwent a PET after the systematic LNE (*n* = 13), additional lymph node metastases were detected in seven patients (53.80%). Distant metastases were suspected in three patients (15.7%) based on PET imaging. The suspicion was confirmed in one patient (peritoneal spread) and excluded in two patients (supra-diaphragmatic lymph nodes). In 13 patients (68.4%), RT procedures were altered due to findings in PET imaging.

**Conclusion:**

PET-based radiochemotherapy planning may improve control rates by identifying areas of tumor cell spread eligible for dose escalation. False positivity, however, should be excluded in patients with findings that lead to major modifications of the therapeutic strategy.

## Introduction

The routine use of Papanicolaou smears in screening examinations as well as the wide availability of vaccination against human papillomavirus have reduced the incidences of cervical cancer in industrialized nations [1, 2]. Nevertheless, cervical cancer is one of the most common types of gynecological cancer with an annual incidence of around 13,000 cases in the United States and represents a major global health burden [1, 2]. Early-stage cervical cancer is treated by surgery, advanced stages by primary radio-(chemo-) therapy [3, 4].

As of recently, PET-CT staging for cervical cancer stages II–IV has been included in the NCCN guidelines [4]. PET-CT is a non-invasive imaging method combining the morphological findings provided by CT with metabolic information. To acquire this information, the patient is injected with the glucose analog 2-fluoro-2-deoxy-D-glucose that is labeled with a positron emitter. Areas with high uptake correspond to sites with high glucose metabolism such as inflamed tissue or tumor cells [5].

In Germany, primary radio-(chemo-) therapy is the treatment of choice for stages IIB and higher and is recommended by the NCCN guidelines even in Stage I if the primary tumor diameter exceeds 4 cm [3]. The combination of external beam therapy (EBRT) with concurrent platinum-containing chemotherapy and brachytherapy with cumulative Point A doses beyond 80–85 Gy is essential for treatment success. The target volume is highly dependent on the primary tumor extension and nodal involvement. It always includes the gross disease, parametria, the uterosacral ligaments as well as the internal, external, obturator, and presacral lymph nodes. To detect lymph node metastases and define the cranial border of the treatment volume, radiochemotherapy should be preceded by a pelvic and paraaortic lymphadenectomy. The common iliac and paraaortic nodes should be covered in patients with a high risk of or confirmed common iliac or paraaortic involvement [4]. A local dose increase to involved unresected nodes may improve disease control. To optimize target volume delineation, the precise knowledge of involved nodal stages is crucial. This might be achieved by the higher sensitivity and specificity of the PET-CT for the detection of lymph node and hematogenous metastasis [6].

PET imaging is already routinely used in radiotherapy planning for other entities. In small and non-small cell lung cancer, PET-based target volume definition is considered standard of care. Its value in the treatment of Hodgkin lymphoma and recurrent prostate cancer is constantly evolving. In light of this knowledge, we intended to implement PET imaging into the treatment planning of cervical cancer [7].

In this study, we report on our first experiences of PET-based radiotherapy planning for cervical cancer at the Technical University Munich.

## Patients and methods

19 consecutive patients with pathologically confirmed cervical cancer who were treated with primary or adjuvant radiochemotherapy at our institution between January 2016 and April 2019 were included in the study. All patients received PET-MRI or PET-CT for radiotherapy planning. This study was approved by the Ethics Committee of the Technical University Munich (Ethics vote number: 141/19-S-SR).

### Radiotherapy (RT) technique

14 patients were treated with primary and 5 patients with adjuvant radiochemotherapy.

External beam radiotherapy was based on 3D-CT simulation creating a conformal Rapid Arc or Intensity-modulated radiotherapy (IMRT) plan.

Patients received 50.4 Gy in daily doses of 1.8 Gy to the primary tumor, parametria, uterosacral ligaments, sufficient vaginal margin from the gross disease (if present), and pelvic lymph node stations in analogy to the RTOG contouring atlas for gynecological cancers. For patients with positive paraaortic lymph nodes, the RT field was extended to Th12/L1 to cover the paraaortic lymph nodes. Involved nodes plus an additional margin of 0.3–0.7 cm received a simultaneous integrated boost with daily doses of 2.0–2.2 Gy to 56.0–61.6 Gy. In some cases, a percutaneous boost was applied to areas of parametrial invasion or vaginal metastases, if present. Radiotherapy was delivered using a linear accelerator with 6/15 MV photons.

For patients primarily treated with radiochemotherapy, brachytherapy was delivered in four fractions of 7 Gy each using an intracavitary applicator set and an Iridium-192 source (GammaMedPlus, Varian Medical Systems). In one patient with primary radiochemotherapy, brachytherapy could not be performed due to the infiltration of the bladder. This patient was treated with salvage hysterectomy after radiochemotherapy.

The chemotherapy protocol consisted of up to six cycles of cisplatin 40 mg/m^2^ in a weekly schedule during EBRT.

### PET imaging

Contrast-enhanced 2-Fluor-Desoxyglucose PET imaging was obtained on a PET-CT (Biograph mCT scanner, Siemens Medical Solutions, Germany) or integrated whole-body MRI (Siemens biograph mMR, Siemens Medical Solutions, Germany) after the intravenous injection of FDG. The median activity of 18F-FDG was 326 MBq (range 200–414 MBq) and the median interval between injection and start of PET acquisition (“uptake time”) accounted for 69 min (range 60–127 min). The examined field extended from the skull base to the proximal femoral.

The results stated in this study were derived from medical reports on PET-CT/MRI. The PET/MRI images were generated with Siemens Biograph mMR and T1VIBE Dixon for attenuation correction. The PET/CT images were generated with Siemens Biograph mCT. The quantitative evaluation of the attenuation-corrected image data is carried out via SUV calculation. The reporting is carried out on approved diagnosis monitors using the SyngoVia (Siemens Healthineers, Germany) evaluation program or Sectra IDS 7 (Sectra AB, Linköping, Sweden). The SUV values are determined with SyngoVia. PET lesions were defined as lesions of cervical cancer if this was stated as the most likely cause for PET positivity in the medical report. As part of the routine procedure, all PET-CT/MRI are interpreted by two nuclear medicine physicians, one of which has completed the board exam and has at least 5 years of experience as well as one board examined radiologist with at least 5 years of experience. Interpretation of the results is generally done in consensus between the nuclear medicine physician and the radiologist. The medical reports were not all written and interpreted by the same physicians.

PET-MRI was used for RT planning in 13 patients, PET-CT in 6 patients. The decision whether PET-CT or PET-MRI was conducted was based on the availability of the methods with a preference for PET-MRI. In all patients who underwent primary RCT and PET-CT, an additional MRI was present for radiotherapy planning.

### Evaluation of acute toxicity

Information on acute toxicity was extracted from documentation during the RT procedure and the patients’ medical records for genitourinary, dermatological, vaginal, and gastrointestinal toxicity.

### Statistical evaluation

Continuous data were expressed as means ± standard deviation or median and range, categorical data as frequency counts or percentages. Follow-up time, overall survival (OS), and recurrence rates were calculated by the Kaplan–Meier method.

Recurrence was calculated from the start of radiotherapy until the date of tumor recurrence. For the evaluation of OS, the time interval between the start of radiotherapy to the date of death or the last contact was calculated.

## Results

### Patients

Median age at diagnosis was 53 years (range 35–81 years). Radiotherapy was completed in all patients as planned. The number of chemotherapy cycles administered was six in five patients, five in eight patients, four in five patients and three in one patient.

### Primary tumor

Based on PET-MRI, previously unknown primary tumor extensions were suspected in three patients receiving primary radiochemotherapy. Tumor locations were parametrial and vaginal invasion in three and two cases, respectively. Whether parametrial invasion was present remained unclear in all three cases. A vaginal metastasis was confirmed in one case after careful gynecological re-examination prompted by PET findings. In one patient, the vaginal extension could be excluded using PET imaging.

In postoperative patients, residual tumor at the vaginal vault was suspected in two cases based on PET. One patient had pT4 R1 disease, the other one pT2b R0 disease. In neither of the cases, macroscopic residual tumor could be confirmed clinically.

### Nodal involvement

15 patients underwent a pelvic and paraaortic lymphadenectomy (LNE). Four patients did not undergo LNE due to complications during surgery in one patient, a radiologically suspected paraaortic disease in one patient, and patient’s refusal in one patient.

**Fig. 1:**
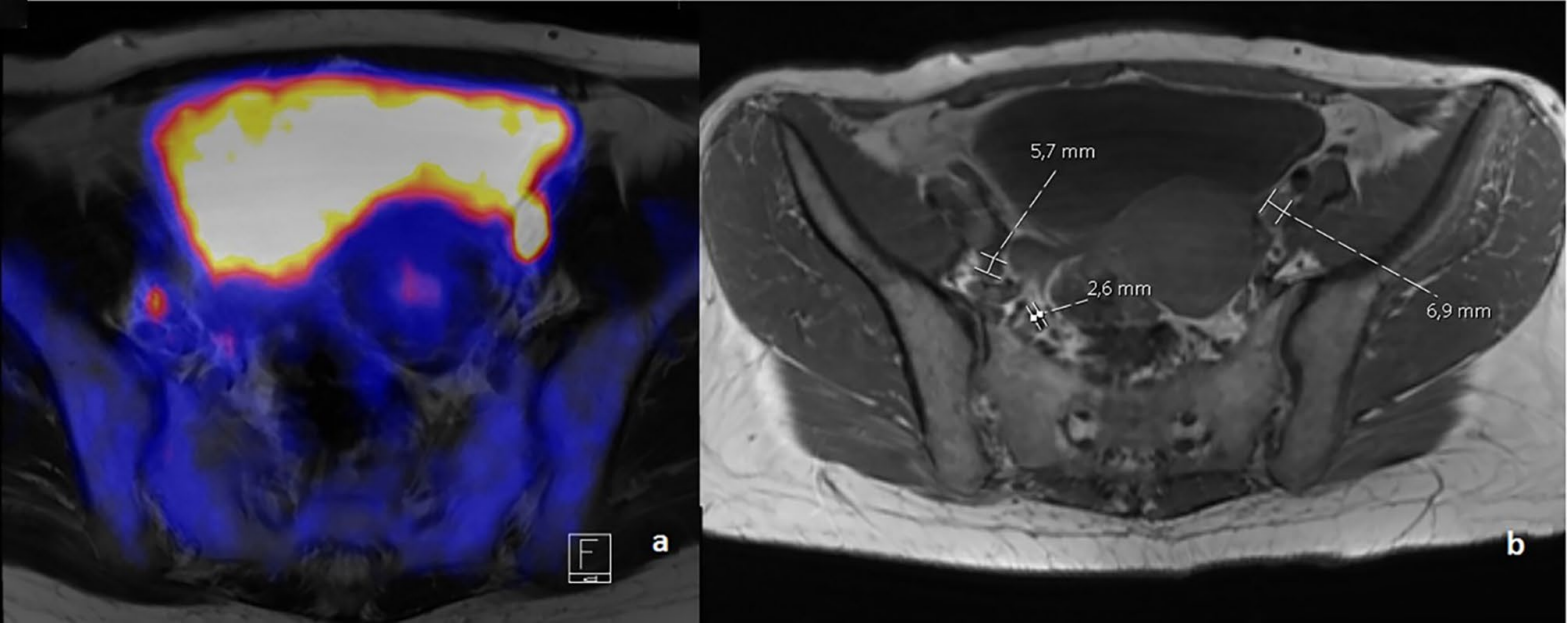
Findings in a patient with preoperative PET-MRI. PET-positive nodes corresponded to histologically involved nodes. **a** PET image **b** corresponding MRI image

In patients who were surgically staged, a mean of 30.7 (± 15.2) nodes were removed. PET was obtained preoperatively in 2 patients and postoperatively in 13 patients. In one of these patients, lymph node metastases were found at anatomical sites corresponding to the PET-positive nodes and were the only lymph nodes of 39 nodes removed containing tumor cells (Fig. 1). In the other patient, neither primary tumor nor lymph nodes showed any sign of FDG uptake. The pathology report after radical hysterectomy and LNE reported a pT1b1 tumor with 5 involved nodes of 16 removed nodes.

Out of the 13 patients who received a PET after the systematic LNE, additional lymph nodes were found in 7 patients (53.8%) with a total of 1–3 lymph nodes per patient. Out of the nine patients with confirmed pathological lymph node metastases, six patients (66.7%) had additional PET-positive nodes with a mean of 1.8 per patient (± 0.8), three did not (33.3%). Out of the four patients without positive lymph nodes in the surgical specimen, additional nodes were identified by PET in two patients (50.0%; mean: 1.5 nodes).

### Metastases

Additional metastases were suspected in three patients (15.8%). The metastatic spread was confirmed in one patient (33.3%) and excluded in two patients (66.6%).

**Fig. 2:**
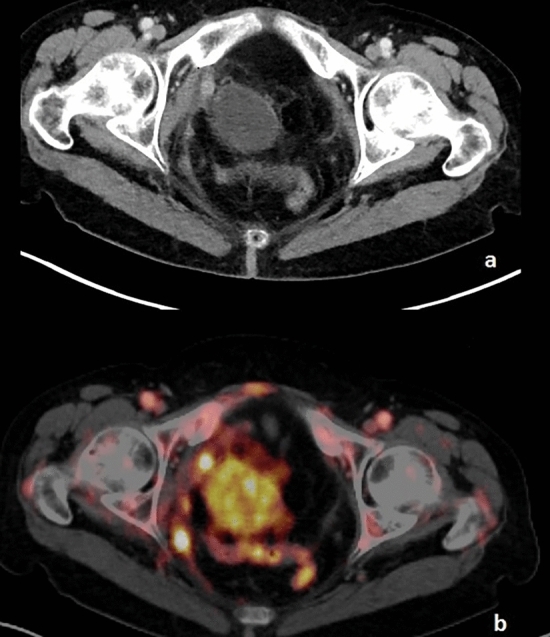
Patient with peritoneal carcinomatosis. **a** CT image **b** corresponding PET Image

In one patient with a large incompletely resected primary tumor and insufficient lymph node dissection (pT4 pN0 (0/1) R1 G3), paraaortic lymph node metastases and peritoneal metastases were suspected in PET-MRI (Fig. 2). Since the operative report did not report peritoneal spread, the RT procedure was conducted without taking a suspicion of peritoneal metastases into account. During follow-up, however, the peritoneal spread was confirmed by follow-up imaging.

**Fig. 3:**
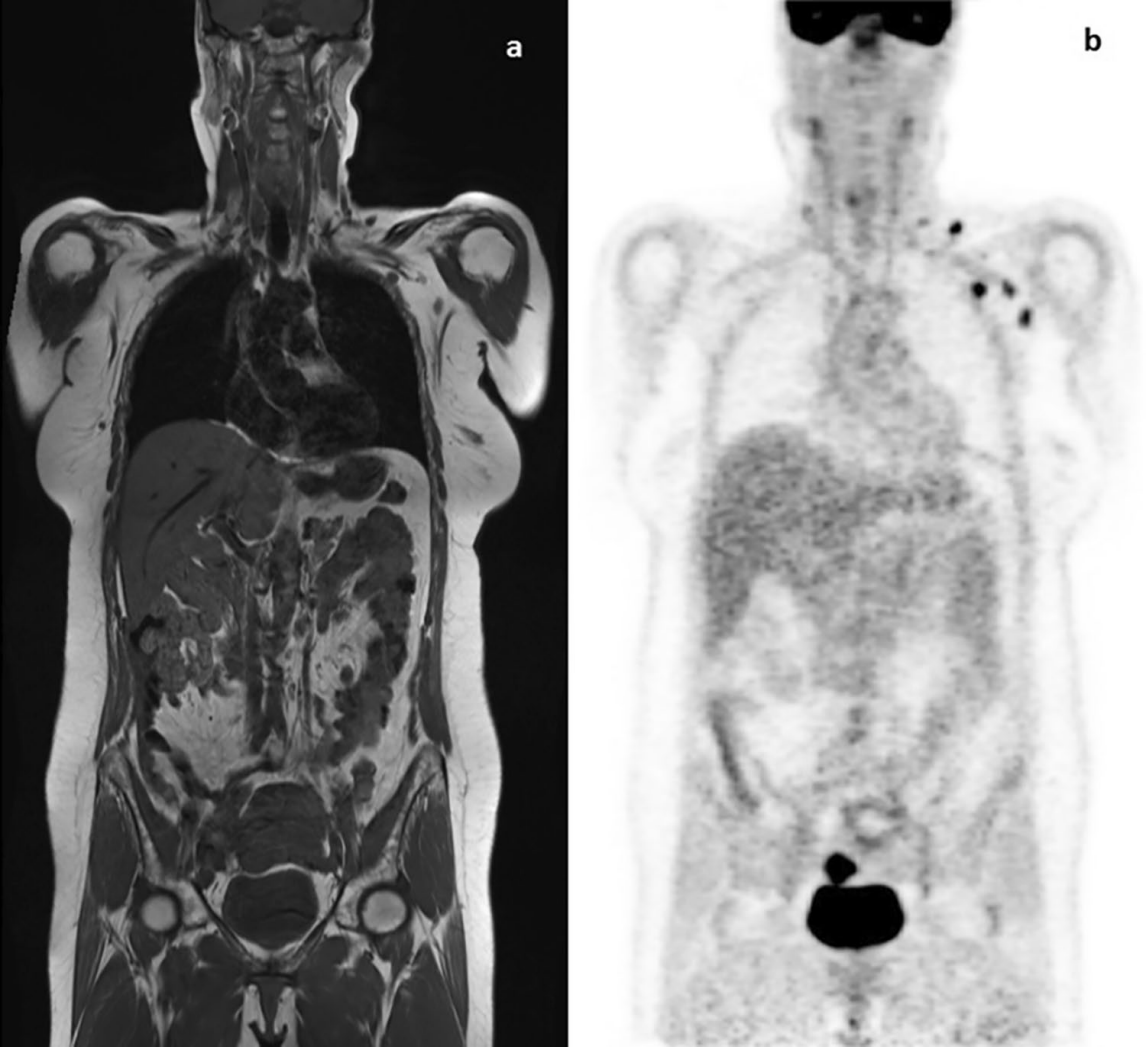
Suspected axillary lymph node metastases that were not confirmed histologically and in follow-up imaging. **a** MRI image **b** corresponding PET image

In one patient, intended for primary radiochemotherapy due to histologically confirmed pelvic and paraaortic lymph node metastases (tumor stage: cT2b pN1 (12/44) pM1 (LYM)), axillary and supraclavicular lymph node metastases were suspected in PET-MRI (Fig. 3). Due to the unusual location for lymph nodes metastases, a clinical breast examination and ultrasound were performed to rule out a secondary cancer. As a vaccination had been performed 2 days before the PET in the corresponding arm and the guided vacuum biopsy did not show signs of tumor cells, the RT procedure was carried out as planned. In the follow-up evaluation 6 weeks after RT, lymph nodes were not enlarged in the performed CT.

**Fig. 4:**
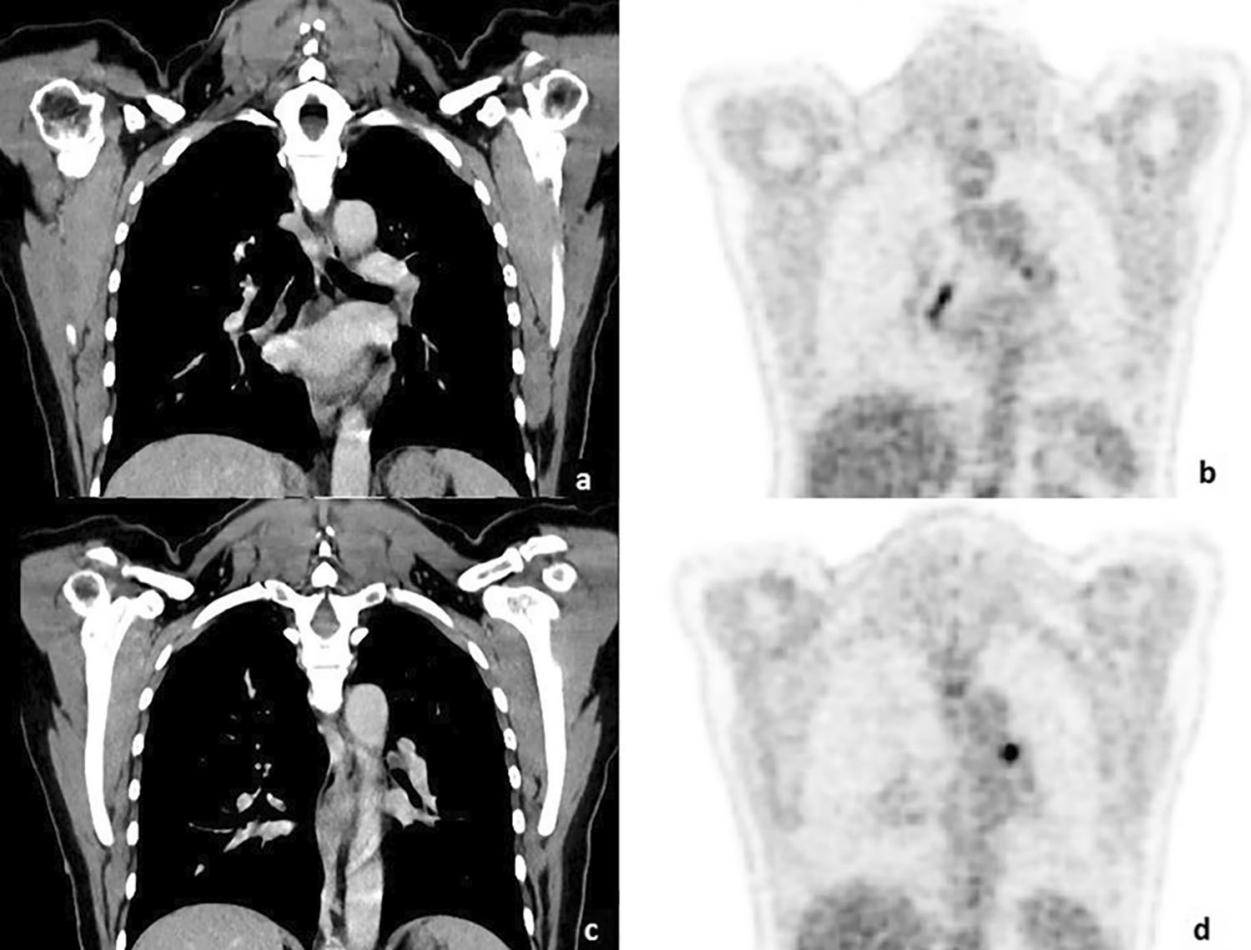
Suspected hilar lymph node metastases; not confirmed in imaging follow-up. **a** CT imaging of LN in a coronary slice **b** PET imaging corresponding to (**a**). **c** CT imaging of LN in a different coronary slice of the same patient. **d** PET imaging corresponding to (**c**)

In one patient planned for primary radiochemotherapy without surgical lymph node staging due to comorbidities, pelvic and hilar lymph node metastases were suspected in the absence of paraaortic lymph node metastases (tumor stage: cT3b cN1 M0, G3-4; Fig. 4). Primary radiochemotherapy was performed without taking into account the potential hilar manifestation. During follow-up, a PET-CT was performed 1 year after the completion of RCT without suspicion of tumor manifestation, particularly without increased tracer uptake in the hilar lymph nodes.

### Changes in FIGO stage by PET

Applying the 2019 modification of the FIGO staging system [8], an upstaging took place in five patients (26.3%). Two patients were reclassified to a FIGO stage IIIC1 due to PET-positive lymph node metastases after a pN0 status in surgical staging. In three patients with initial FIGO stage IIIC1, IIIC2 and IVA, stage IVB was suspected based on PET imaging. However, the metastatic disease could only be confirmed in one of the three patients as mentioned above.

### Changes in RT procedure

In 13 patients (68.4%), RT procedures were altered due to findings in PET imaging. The majority of changes in the RT procedure were the integration of a simultaneous boost to the area of lymph node metastases in 12 cases. An additional boost of the vaginal metastases was performed in one patient.

### Toxicity

There was no case of grade 3 or higher dermatological, gastrointestinal, or genitourinary toxicity according to CTCAE version 6.0. Two patients with vaginal metastases reported grade 3 vaginal mucositis. Acute vaginal toxicity did not exceed grade 3 in other patients.

### Follow-up

The mean follow-up was 18.3 months (± 2.8 months). The mean overall survival was 33.6 months (± 3.6 months). At the time of analysis, four patients had developed distant metastases, of which two patients died from progression. In one of the diseased patients, a suspicion of peritoneal carcinomatosis was present in the initial PET-MRI. At first follow-up 6 weeks after the completion of RT, the initially PET-positive lymph nodes and in-field peritoneal manifestations were no longer detectable. However, new peritoneal manifestations were found out-of-field. Locoregional lymph node recurrence occurred in three patients, none of the patients developed local recurrence. 1-year PFS was 83.1%.

## Discussion

In this study, we report our experiences of PET-CT/MRI-based radiotherapy planning in cervical cancer. PET imaging altered the radiotherapy procedure in 68% of our patients mainly through the integration of additional boost volumes. On the other hand, distant metastases were falsely suspected in two patients based on the PET findings. Two of our patients received PET imaging before surgical staging. In one patient, the involved nodes corresponded to the PET-positive ones. The other patient was PET negative for primary cancer and involved nodes.

PET-CT scan has recently been included in the NCCN guidelines for cervical cancer as the preferred staging method in clinical stage II or higher disease and may be considered for stage IB1/2. In this study, we report our experiences with PET-CT/MRI-based radiotherapy planning which was first started in 2016 and became the standard method for radiotherapy planning at our institution in 2018.

The findings of the PET imaging altered the dose prescription and target volume in approximately 70% of patients, mainly through the definition of simultaneous integrated boost volumes for presumed additional or newly diagnosed lymph node manifestations. In one patient, the PET imaging prompted a careful gynecological re-examination leading to the discovery of a vaginal metastasis that was subsequently treated with an increased radiation dose. The identification of areas with macroscopic tumor spread may pave the road towards dose escalation. This is of special interest in the case of lymph node involvement. Sophisticated brachytherapy techniques have led to local tumor control rates of approximately 90% even at higher tumor stages in recent years with [9, 10].

Thus, local control has overcome regional nodal control which has been reported to approximate 80% [11]. Both marginal and in-field failure contribute to a similar extent to regional control [12]. The majority of nodal out-of-field recurrences occur immediately above the irradiation field. These findings suggest a deficiency in both target volume definition and radiation dose [12]. Higher radiation doses and an extension of the radiation field improve nodal control [10, 13]. However, toxicity has to be weighed against higher control rates as the radiation dose in the small pelvis is limited by the dose constraint of the small bowel. A dose–escalation above 50 Gy in this organ at risk is only feasible in small areas. Simultaneous integrated boost concepts have been described by some studies using doses between 54 and 69.4 Gy with acceptable toxicity. To properly define the involved nodes, particularly with diameters below 1 cm, combined PET and conventional imaging provides higher sensitivity and specificity than CT or MRI alone [6]. As a consequence, it represents the obvious choice to assist in SIB target volume definition and may contribute to control rates by providing a possibility to overcome the limitations mentioned above. Since associations between standard uptake value and nodal control rates have been reported even PET-based dose definition in pelvic nodes seems possible in the future [11].

However, the false-negative rate for paraaortic nodes has been shown to lie around 12% in two prospective trials [14, 15] signifying dramatic undertreatment for these patients if solely PET-based radiotherapy planning would have been applied. Hence, a combination of surgical staging and PET imaging seems to provide higher diagnostic accuracy for paraaortic nodes over PET imaging alone.

The false-positivity rate has not been evaluated in prospective trials but has been described to range around 82% in a retrospective study [16]. In our study, no case of PET-positive paraaortic lymph node manifestation without pathologically positive nodes has been recorded in patients with surgical staging. PET-positive supra-diaphragmatic lymph nodes, on the other hand, were identified in two patients. In both cases, the lymph node manifestation turned out to be reactive. As supra-diaphragmatic nodes are classified as metastatic manifestations, the PET findings would have deprived these patients of a curative approach. PET imaging should, therefore, not be applied as the sole diagnostic to identify lymph node metastasis. Especially in patients with findings that lead to a major modification of the therapeutic approach (i.e. metastatic or paraaortic lymph node discovery), a histological confirmation should be attempted.

There are certain limitations to our study. The low number of patients treated with PET-based planning and the short follow-up time do not allow conclusions regarding the outcome. Furthermore, the retrospective nature of this study led to the inclusion of patients with different sequences of surgical staging and PET imaging, with the majority of patients receiving PET before surgery. It is, therefore, unclear whether the post-surgical PET-positive nodes represent tumor manifestations. Nevertheless, we provide evidence that PET-based dose escalation is well tolerated and that supra-diaphragmatic PET positivity should not automatically lead to a palliative approach.

## Conclusion

PET-CT-based radiochemotherapy planning may improve control rates by identifying areas of tumor cell spread eligible for dose escalation. False positivity, however, should be excluded in patients with findings that lead to major modifications of the therapeutic strategy. Particularly, suspected metastases should be confirmed histologically.

## Data Availability

The datasets generated and/or analyzed during the current study are not publicly available due to privacy regulations in the ethics approval but are available from the corresponding author on reasonable request.
